# Semiconductor Quantum Dots as Target Analytes: Properties, Surface Chemistry and Detection

**DOI:** 10.3390/nano12142501

**Published:** 2022-07-21

**Authors:** Jesús Sanmartín-Matalobos, Pilar Bermejo-Barrera, Manuel Aboal-Somoza, Matilde Fondo, Ana M. García-Deibe, Julio Corredoira-Vázquez, Yeneva Alves-Iglesias

**Affiliations:** 1 Coordination and Supramolecular Chemistry Group (SupraMetal), Department of Inorganic Chemistry, Faculty of Chemistry, Institute of Materials (iMATUS), Universidade de Santiago de Compostela, Avenida das Ciencias s/n, 15782 Santiago de Compostela, Spain; matilde.fondo@usc.es (M.F.); julio.corredoira.vazquez@usc.es (J.C.-V.); yeneva.alves.iglesias@usc.es (Y.A.-I.); 2 Trace Element, Speciation and Spectroscopy Group (GETEE), Department of Analytical Chemistry, Nutrition and Bromatology, Faculty of Chemistry, Institute of Materials (iMATUS), Universidade de Santiago de Compostela, Avenida das Ciencias s/n, 15782 Santiago de Compostela, Spain; pilar.bermejo@usc.es (P.B.-B.); m.aboal@usc.es (M.A.-S.)

**Keywords:** QDs, size, composition, properties, surface chemistry, detection

## Abstract

Since the discovery of Quantum Dots (QDs) by Alexey I. Ekimov in 1981, the interest of researchers in that particular type of nanomaterials (NMs) with unique optical and electrical properties has been increasing year by year. Thus, since 2009, the number of scientific articles published on this topic has not been less than a thousand a year. The increasing use of QDs due to their biomedical, pharmaceutical, biological, photovoltaics or computing applications, as well as many other high-tech uses such as for displays and solid-state lighting (SSL), has given rise to a considerable number of studies about its potential toxicity. However, there are a really low number of reported studies on the detection and quantification of QDs, and these include ICP–MS and electrochemical analysis, which are the most common quantification techniques employed for this purpose. The knowledge of chemical phenomena occurring on the surface of QDs is crucial for understanding the interactions of QDs with species dissolved in the dispersion medium, while it paves the way for a widespread use of chemosensors to facilitate its detection. Keeping in mind both human health and environmental risks of QDs as well as the scarcity of analytical techniques and methodological approaches for their detection, the adaptation of existing techniques and methods used with other NMs appears necessary. In order to provide a multidisciplinary perspective on QD detection, this review focused on three interrelated key aspects of QDs: properties, surface chemistry and detection.

## 1. Introduction

Quantum dots (QDs) mostly consist of semiconductor inorganic materials (e.g., CdTe, CdSe, CdS or PbSe) with diameters typically in a range of 2–10 nm, corresponding to 100 to 100,000 atoms within the nanoparticle volume. Their composition and extremely small size are responsible for their unique optical properties (such as their absorption and emission maximum wavelengths, as well as their bandwidths) and electrical properties, such as their conductivity and charge carrier type, i.e., holes or electrons.

QDs yield emission wavelengths spanning from the infrared to the ultraviolet region of the electromagnetic spectrum. QDs with diameters of 5–6 nm emit longer wavelengths if excited by UV light, while smaller ones with diameters in a range of 2–3 nm emit shorter wavelengths. Since electrons within QDs are confined to discrete energy levels, which are governed by the particle size (the smaller the particle size, the larger the band gap) [1], the emission wavelengths are tuneable by changing the size of QDs and, of course, its chemical composition. As occurring with the optical properties of QDs, electrical properties can be also highly tuneable, which makes them very interesting for a number of applications [2,3,4].

QDs are among the most investigated materials because of their biomedical, pharmaceutical, biological, photovoltaics and computing applications, as well as due to many other high-tech usages, such as displays and solid-state lighting (SSL). Most of the QDs used in analytical applications are core/shell structures [5] rather than conventional simple QDs. Core/Shell QDs (CS QDs) are composed of a semiconducting core material surrounded by a shell of a distinct semiconducting material. A critical advantage of CS QDs over simple QDs is that CS QDs can improve properties or even display some other ones, which cannot be shown by simple QDs.

The possible release into the solution of toxic or poisonous ions such as Cd^2+^, Pb^2+^, Se^2−^ or Te^2−^, even at low concentrations, has raised concerns about the potential impact of QDs both on human health and environment. However, its toxicity has been questioned because of the discrepancy between results obtained from in vitro and in vivo studies on CdSe QDs [6]. While the toxicity of CdSe has not been unquestionably demonstrated using in vivo experiments in animal models, in vitro experiments have shown that QDs can cause cytotoxicity by releasing Cd^2+^ ions into solution and by generating free radical species. Therefore, a deeper knowledge of the surface chemistry of QDs is essential for a better comprehension of how they may interact with biological systems such as proteins, peptides, oligonucleotides and humic acids or with synthetic organic substances, such as thiol-based ligands. Both natural and synthetic species can bring electron density within the coordination sphere of the metal ion, with the destabilisation of bonds in the surface lattice of QDs, leading to the release of ions into the solution. Hence, the main concepts of surface coordination chemistry should be briefly reviewed for a better understanding of the QDs reactivity, either with a view to its stability, toxicity or eventually its detection.

By contrast, carbon-containing NMs, such as carbon QDs (CQDs) or graphene QDs (GQDs), do not present potential toxicity, as they lack toxic ions. Moreover, these QDs display a wide variety of applications, such as bioimaging, sensors, photovoltaics, etc. [7,8]. Regarding the synthesis of carbon-based NMs, in theory, it can be as expensive as the synthesis of any other NMs. However, economical and facile technologies for the obtention of carbon-based materials (QDs among them) from biological resources are currently well-documented. For instance, the recycling of a waste material such as crayfish-shell derivatives [9] or the use of fungal mycelium-based materials [10] can be the base to prepare NMs. Undoubtedly, these topics open a wide variety of promising possibilities for the future of nanotechnology.

A current challenge for researchers in the field of analytical chemistry is to develop innovative approaches or to adapt existing analytical methods used with NMs to detect and quantify QDs. In addition, methodology should be applicable to track QDs quantitatively not only in homogeneous samples but also in complex samples and at realistic concentrations. Until now, there is a remarkably low number of studies reporting the detection and quantification of QDs, including ICP–MS and electrochemical analysis, which are the most common quantification techniques used for this purpose. The quantification of graphene QDs in aqueous samples using its intrinsic fluorescence emission has been reported by Benítez-Martínez and Valcárcel [7]. Lewinski et al. [11] reported the quantification of water solubilised CdSe/ZnS QDs in a small planktonic crustacean by using ICP–MS measurements in combination with confocal fluorescence microscopy. Medina-Sánchez et al. [12] presented the electrochemical detection of CdS by using a hybrid polydimethylsiloxane-polycarbonate (PDMS–PC) microfluidic platform with an integrated screen-printed electrode (SPE).

During the past ten years, several review articles have focused on the properties [2,13,14,15], applications [2,3,4,13,14,15,16,17,18,19,20,21,22], toxicity [6,15,21,22,23,24,25,26,27,28,29,30,31,32,33,34,35], synthesis [14,16,17,18,22,29,36], characterisation [16,17,37,38,39,40] and functionalisation [29,32,41,42,43] of QDs, but to the best of our knowledge, there are no reviews reporting detection methods for QDs. The aim of this paper is to review the essential knowledge on QDs as target analytes. We will focus on three interrelated key aspects of QDs: the effect of size and composition on properties, surface chemistry and detection from a multidisciplinary perspective. Moreover, the used analytical methods suitable for the detection and characterisation of NMs showing potential capabilities to be used with QDs are considered.

## 2. The Effect of Size and Composition on Properties of QDs

The unique optical and electrical properties of QDs are between that of a bulk material and discrete molecules. The keys to understanding the properties of QDs are both their small size and their chemical composition.

**Figure 1:**
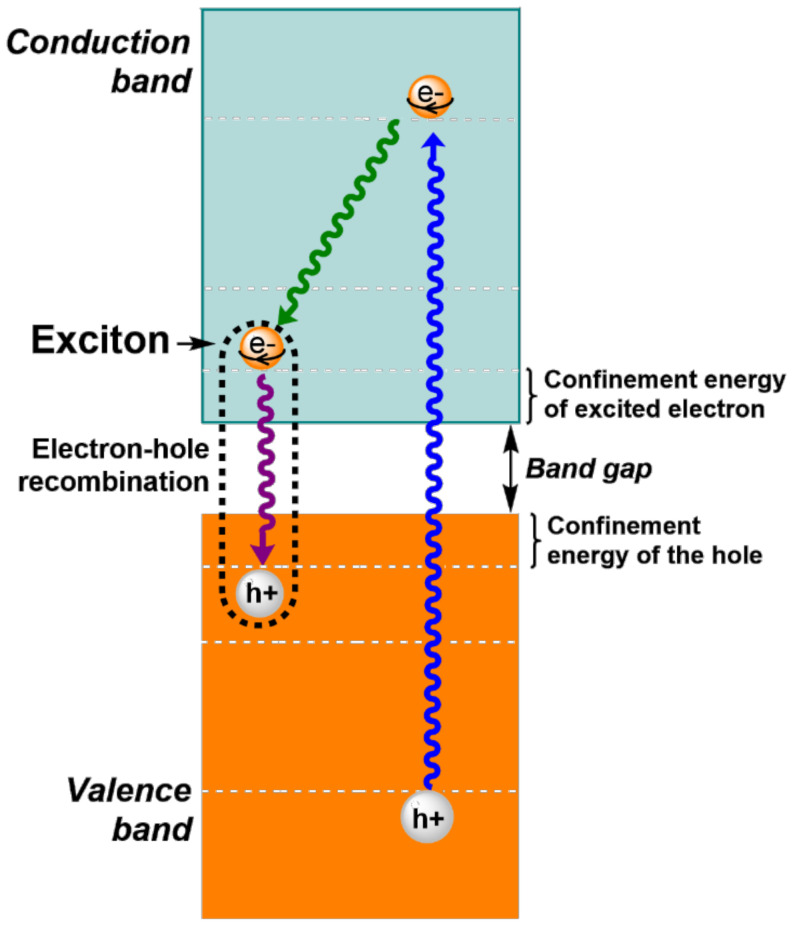
Formation of the exciton from the electron-hole pair and subsequent electron-hole recombination.

The particle size of QDs is typically in the range of 2–10 nm in diameter [44], although QDs with 2–50 nm diameters and even larger have been reported. However, the smallest ones display the most interesting properties, showing drastic differences with larger NMs in optical absorptions, exciton binding energies and the decay of the exciton. An exciton (electron-hole pair) is created when an electron leaves the valence band and enters the conduction band due to the excitation of QDs (Figure 1). This gives rise to an empty state in the valence band, i.e., a hole, which can be thought as a positive charge. In excitons, a weak columbic force of attraction exists between the hole and electron pair, and the average separation distance between them is known as the exciton Bohr radius. The exciton Bohr radius (α_B_) of a NM can be calculated using the following equation [45], where h is the Planck’s constant, ε is the dielectric constant, e is the electron charge, m_e_* is the effective mass of the electron, and m_h_* is the effective mass of the hole.

$$\alpha_{B}=\frac{h^{2}\cdot \varepsilon }{e^{2}}\left(\frac{1}{m_{e}^{*}}+\frac{1}{m_{h}^{*}}\right)$$

After excitation, which causes the formation of the exciton, both electrons and holes rapidly move to energy levels near the confinement energies of excited electron and hole, respectively, and then electron-hole pair recombination occurs. The released energy can be calculated as the sum of the following three terms: the confinement energies of the excited electron and hole, the band gap energy and the bond energy of the exciton.

It must be noted that, in bulk materials, the particle sizes are much larger than the exciton Bohr radius such that the energy levels form continuous bands of energy rather than discrete energy levels. As a result, the conduction band and valence band in bulk materials are separated by a relatively small band gap, if compared to the band gap of QD materials. In contrast, as the size of QDs is less than twice the bulk exciton Bohr radius [45], the energy levels are discrete, resulting in a larger band gap in comparison with that of bulk materials. Therefore, the band gap increases with decreasing particle sizes, and inversely, an increase in size leads to a lower band gap. The optical band gap (i.e., the threshold energy for photons to be absorbed) of simple QDs can be determined from UV–Vis absorption data using the Tauc plot method [46], extrapolating the linear part of the plot to the *x*-axis. Typically, a Tauc plot shows hν on the *x*-axis and (α·hν)^1/γ^ on the *y*-axis [47], where α is the absorption coefficient of the NM (i.e., absorbance/thickness of sample), h is the Planck constant and ν is the frequency of the photon (i.e., c/λ_abs_). The γ factor depends on the nature of the electron transition, and it is equal to 1/2 or 2 for the direct and indirect transition band gaps, respectively. The Tauc method assumes that the energy-dependent absorption coefficient α can be expressed by the following equation, where *E*_g_ is the band gap energy, B is a constant and the remaining terms of the equation have been already indicated.

$${\left(\alpha \;\cdot \;h\nu \right)}^{1/\gamma }=B\left(h\nu \;-\;E_{g}\right)$$

The band gap is a very important parameter of QDs, since it determines some of their properties. The size-dependent band gap of quantum dots can be explained in terms of the quantum confinement effect [1,48]. In QDs, excitons are under a three-dimensional confinement that results in the quantisation of the electron and hole energy levels. Semiconductor materials of size less than double the bulk exciton Bohr radius undergo quantum confinement. As examples, the quantum confinement effect will be observed in CdS, CdSe and CdTe NMs when their particle diameters are less than ~5.8 nm, 11.2 nm and 20.0 nm, respectively. Thus, bulk exciton Bohr radius and, by extension, composition determine the size from which a material exhibits quantum confinement effects and, consequently, unique optical and electronic properties of QDs. Since electrons within QDs are confined to discrete energy levels, the wavelengths associated with the formation and recombination of excitons (i.e., the UV–Vis absorption and fluorescence emission maximum wavelengths of QDs, respectively) are tuneable by changing the particle’s size. With decreasing size, the wavelengths of fluorescence and absorption decrease. Table 1 shows the values of the maximum wavelength for fluorescence emission and visible absorption spectra corresponding to CdSe QDs with particle diameters in the 1.8–7.3 nm range, as a representative example of the effect of size on fluorescence emission and absorption in the visible range [49].

The range over which the band gap energy and, therefore, wavelength of fluorescence and absorption can be tuned by quantum confinement depends not only on the size particle but also on the composition of the material. Band gap energy (*E*_g_) varies with the composition of the material following two trends. (i) *E*_g_ decreases when proceeding down a group in the periodic table of the elements. This is because *E*_g_ is related to the energy difference between bonding and antibonding molecular orbitals, which decreases as the principal quantum number (*n*) increases. (ii) The band gap’s energy increases with the increase in ionicity in isoelectronic compounds. This can be understood by having in mind that an increase in electronegativity differences between atoms (Δχ) lead to an increase in the energy difference between bonding and antibonding orbitals.

QDs mostly consist of semiconductor inorganic materials, which are usually prepared with elements belonging to groups 12–16 of the periodic table (binary II–VI type QDs) and 14–16 (binary IV–VI type QDs). Although less researched, there are many others, such as metal-free elemental QDs (unary IV type QDs) and, of course, inorganic QDs, such as binary III–V type QDs, ternary I–III–VI type QDs and quaternary I–II–IV–VI type QDs. All these materials, both bulk and QD forms, have band gap energies ≤4.0 eV and, therefore, are semiconductors. Band gap energy for a few materials, which are commonly used to prepare QDs, are provided in Table 2 [14,50,51,52]. An appropriate selection of both size and composition can lead to fluorescence wavelengths in both visible (380–750 nm) and near-infrared (750–2500 nm) ranges.

**Figure 2:**
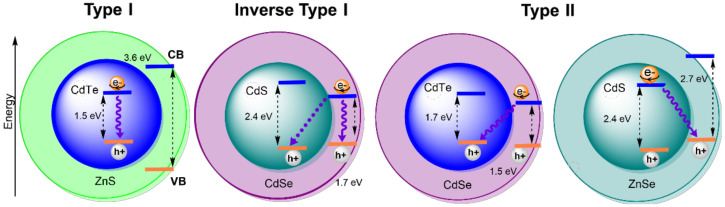
Schematic representation of the three main types of CS QDs: Type I, Inverse type I and Type II, of which CdTe/ZnS, CdS/CdSe and CdTe/CdSe, as well as CdS/ZnSe, are examples. Band gap energies (*E*_g_) for the semiconductor materials used as examples are indicated. Electron-hole recombination is indicated by a wavy arrow.

Among QDs, the so-called core/shell QDs (CS QDs) exhibit improved chemical and physical properties over conventional simple QDs due to a shell of a second semiconductor layer material that surrounds the core [5]. Furthermore, the shell provides protection against environmental changes and photo-oxidative degradation. CS QDs can be categorised according to the difference between the band gap energy (*E*_g_) of the core and the shell into the following three main groups (Figure 2) [5].

Type I: The band gap of the core material is smaller than that of the shell (*E*_g (shell)_ > *E*_g (core)_), leading to the localisation of both electrons and holes in the core, which occurs in CdTe/ZnS QDs. Reverse type I: The band gap of the shell material is smaller than that of the core (*E*_g (core)_ > *E*_g (shell)_), which leads to the localisation of both electrons and holes in the shell (e.g., CdS/CdSe QDs). Type II: The bandgap of both core and shell materials has a similar value (*E*_g (core)_~*E*_g (shell)_); this leads to the localisation of the electrons in the shell and the holes in the core or vice versa, as occurs in CdTe/CdSe QDs and CdS/ZnSe QDs, respectively. Type I CS QDs are the most common in bioanalytical applications since this configuration provides the best confinement of the exciton and the highest rates of radiative electron-hole recombination (i.e., brightness of the photoluminescence).

QDs are frequently coated with an external layer or protective shell responsible for stability, solubility and functionalisation. This is commonly achieved using surface ligands. Surface ligands also enable control over the stability of QDs dispersions in polar or nonpolar solvents via both steric and electrostatic mechanisms. Organic ligands with hydrophilic tails, such as –COOH, –OH, –SH, etc., are chosen to produce water soluble QDs. By contrast, ligands featuring a hydrocarbon tail, such as long-chain alkyl phosphines, alkyl phosphine oxides, alkyl amines and alkyl thiols, etc., can afford QDs with higher solubility in nonpolar solvents. The external layer of passivating ligands enhances the stability of QDs by preserving under-coordinated atoms existing on the outermost layer of the core material against chemical processes, such as oxidation and degradation. This is of crucial importance in QDs composed of a cadmium-based core material, such as those used as diagnostic and therapeutic tools in nanomedicine. CdS QDs have been coated with different surface ligands, such as glutathione [53,54] and *N*-acetyl-l-cysteine [53]. Owens et al. [55] synthesised both CdSe and ZnSe QDs protected with several organic ligands such as thioglycolic acid (TGA), thioglycerol (TGL) or glutathione (GSH), with varying thiol-to-metal ratios. Likewise, Gallagher et al. [56] coated CdSe/ZnS QDs with tri-*n*-octylphosphine oxide (TOPO). Many polymer coatings are also suitable as protective shells on the surface of QDs. As examples, we can mention poly-vinyl alcohol (PVA), poly-methyl methacrylate (PMMA), poly-ethylene glycol (PEG) and poly-lactide-co-glycolides (PLGA). In the case of CdSe/ZnS QDs, these have been coated with amphiphilic polymers such as poly(acrylic acid)-octylamine and poly(maleic anhydride-alt-1-octadecene), previous to a further conjugation with methyl-polyethylene glycol-amine [11] or with PMMA [56].

The surface functionalisation of QDs for improved water solubility has the advantage of favouring its detection in aqueous media [54] but with the inconvenience of increasing its potential toxicity. This may be due to the release of Cd^2+^ ions [53,56] and fragments that result from QD weathering [53] or to the generation of reactive oxygen species (ROS). Therefore, a deeper understanding of the toxicity of coated QDs under real-life conditions is more than desirable [11].

## 3. Surface Chemistry of QDs

Because of their small size, QDs have a high ratio of surface atoms to core atoms, which causes the surface atoms to play a decisive role not only in their properties but also in the reactivity of the entire particle. As Table 3 shows, the percentage of surface atoms rapidly increases when the particle size is less than 10 nm. As the particle size reduces to 1 nm, the surface atomic percentage will be approximately 90%, and the atoms are mostly concentrated on the surface of QDs.

The lattice termination on the QD surface has as a consequence that surface atoms are typically undercoordinated, which makes them more reactive than those ones in the core. Although surface atoms are coordinated to passivating ligands, which are commonly used to prevent the agglomeration and growth of QDs, some degree of undercoordination prevails because of the steric hindrance between ligands. This effect has been demonstrated by the spectroscopic studies of the QD’s surface using the following analytical techniques: nuclear magnetic resonance spectroscopy (NMR), Fourier transform infrared spectroscopy (FTIR), extended X-ray absorption fine structure spectroscopy (EXAFS) and Rutherford backscattering spectroscopy (RBS) [57,58,59,60].

**Figure 3:**
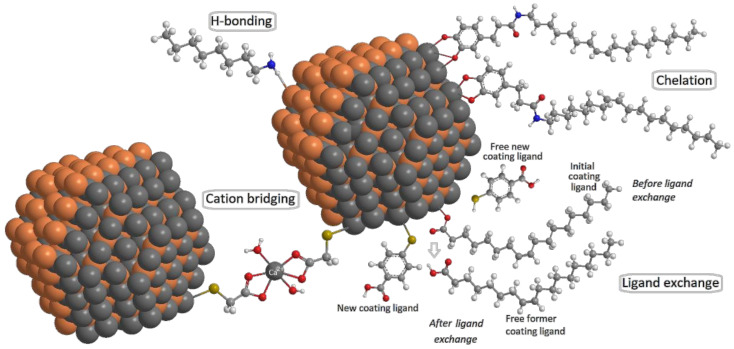
Schematic diagram to illustrate the bonding forms for main adsorption modes of dissolved ligands onto the surface of CdSe QDs (Cd: grey balls; Se: brown balls). Left top: CdSe-octylamine has been used here as an illustrative example of an H-bonding interaction. Right top: The interaction between CdSe and a catechol-based oleylamine shows a couple of modes of chelation. Right-bottom: The desorption of oleic acid from coated QDs and the subsequent absorption of 4-mercaptobenzoic acid illustrates the ligand exchange mode. Left-bottom: The interaction between thioglycolate-capped QDs and Ca^2+^ shows cation bridging mode among particles.

The typical processes in the reaction of dissolved ligands (adsorbates) with nanomaterial surfaces (adsorbents) can be classified into two general types: physisorption and chemisorption [61]. However, it must be noted that no sharp distinction can be made between the forces involved in these interactions, and intermediate cases exist. This is the case of adsorption involving strong hydrogen bonds or weak charge transfer. On the one hand, physisorption (or physical adsorption) is adsorption in which binding energy is typically in the range of 0.010–0.300 eV. Forces such as weak hydrogen bonds, electrostatic interactions, van der Waals interactions, hydrophobic interactions, etc., are involved in physisorption. On the other hand, chemisorption (or chemical adsorption) involves binding energies with typical values in the range of 1–10 eV. The forces involved in chemisorption are valence forces of the same kind as those operating in the formation of chemical compounds, such as the formation of coordinate bonds, or even adsorption involving strong hydrogen bonds. Among the possible chemical adsorption modes of organic adsorbates on the surfaces of NMs, ligand exchange, cation bridging and chelation (Figure 3) are reported.

### 3.1. Interactions between NMs and Surface Ligands

It is noteworthy that several different modes of interaction have been proposed to explain the adsorption behaviour of organic ligands on NM surfaces, but in most cases, more than one interaction is involved. In addition, their relative contributions may vary depending on the chemical nature of the organic ligand, the NM and the solvent. Below, hydrophobic, π–π and electrostatic interactions, as well as hydrogen bonds, cation bridging, ligand exchange and surface anions chelation, are briefly commented upon.

Hydrophobic interactions preferentially occur between the nonpolar regions of organic adsorbates and NMs, similarly to carbon QDs, have nonpolar surfaces [62]. Thus, the aromatic moieties of organic ligands can be weakly adsorbed on carbon nanomaterial surfaces by hydrophobic effects [63]. The weaker adsorption of organic ligands to the carbon NM surface at higher pH values evidences that the ionisation of functional groups decreased hydrophobic effects, showing that this interaction type is pH-dependent.

Regarding π–π interactions, they can occur between organic ligands containing aromatic moieties (or C=C double bonds) and the π electrons of benzene rings on the surface of NMs, such as carbon QDs [64,65]. π–π electron coupling may be influenced by the number of conjugate groups and the relative position of aromatic rings of adsorbates to benzene rings on carbon NM surfaces [66].

When organic adsorbates and nanomaterials are oppositely charged, an electrostatic interaction can exist between them. In fact, the solvation of nanomaterial surfaces by the addition of capping agents used to stabilise bare nanomaterials, or simply because of the point of zero charge (PZC), can lead to charged nanomaterials [67,68]. Likewise, there is a number of organic adsorbates for which their charges are pH-dependent. Accordingly, the net negative charge on the adsorbate increases with high pH values because of the ionisation of acidic groups, whereas the positive charge can increase with reduced pH because of the protonation of basic groups. Therefore, the electrostatic interaction is strongly pH-dependent.

Hydrogen bonds can also occur when organic adsorbates and nanomaterials contain polar functional groups, such as –COOH, –OH, –NH_2_, etc., which have high electronegativities. These groups can act as hydrogen-bonding donors, since they have a highly polar hydrogen atom bonded to an electronegative atom (mostly N, O and F) and, therefore, can establish H-bonds with other atoms with a partial negative charge. At the same time, since N, O and F atoms on polar functional groups have a partial negative charge, they can accept H-bonds from other atoms by sharing their lone electron pairs [69]. Therefore, hydrogen bonding interactions have been reported between organic adsorbates and carbon-based nanomaterials [70]. H-bonding interactions are also strongly dependent on pH, and an increase in pH could lead to the deprotonation of acidic functional groups such as –COOH and –OH moieties, which would reduce the number of H-bonding donors.

With regard to cation bridging or bridging chelation, this interaction occurs when anionic or polar functional groups (typically carboxylate-terminated ligands) become bound to a multivalent metal ion that is adsorbed to a negatively charged NM surface. Zhang et al. [71] studied the stability of thioglycolate-capped CdTe QDs in monovalent and polyvalent electrolytes. They observed that the QDs remained stable even at 150 mM KCl solution while aggregates occurred at relatively low concentrations (≤2 meq L^−1^) of divalent cations. It was concluded that multivalent cations (Ca^2+^ and Al^3+^, etc.) may react with the capping ligands of QDs to form complexes that bridge QDs, which induces the formation of settleable QD flocs. As the cation bridging effect not only occurs between adsorbates and NMs but can also exist between adjacent organic adsorbate interfaces, the multilayer adsorption of organic adsorbates onto NM surfaces could be expected. Meanwhile, the compression of electric double layers by cations and the increase in hydrophobicity of organic adsorbates also promote its adsorption [72].

A process leading to the desorption of original surface ligands is ligand exchange, which can be used during the synthesis of QDs to adsorb new ligands, with the aim of changing their optical properties [73,74,75]. Thus, the substitution can affect certain aspects such as head or tail groups or the chain’s length. Surface ligands can be classified into three types according by the number of donated electrons (2, 1 or 0) into L-, X- or Z-types [74,75]. L-type ligands (Lewis bases) are neutral with two electrons coordinated to surface metal ions, e.g., amines (RNH_2_), phosphines (R_3_P), phosphine oxides (R_3_PO), etc. Anions that offer one electron are often categorised as X-type ligands, such as carboxylates (RCOO^−^), thiolates (RS^−^), halides (Cl^−^), etc., which interact with either metal or nonmetal ions. Finally, Z-type ligands (Lewis acids) usually serve as acceptors of electrons, binding anions on QDs’ surface. Examples of Z–type ligands include CdX_2_, PbX_2_, etc.

Surface anion chelation occurs when the interaction between the surface of the QDs and the organic adsorbate ligand occurs via a direct bidentate interaction [76], as Figure 3 shows. Both fulvic and humic acids, as well as catechols, are good examples of organic ligands with two oxygen atoms of neighbouring phenol groups forming a strong chelating bond to surface metal ion [77,78,79].

### 3.2. Analytical Techniques for Characterising the Interactions between NMs and Surface Ligands

A brief comment about what data can be obtained from some common analytical techniques can be found below. This information can be used to study the interaction between NMs and surface ligands. It is rather unusual that researchers obtain data by just one analytical technique but from two or more of them given the complementary information that those techniques provide.

#### 3.2.1. Nuclear Magnetic Resonance (NMR)

NMR spectroscopy allows characterisation with non-destructive perturbations of the sample, providing rapid and remarkable information about the chemical environment of the analysed nuclei. The combination of both 1D (^1^H, ^13^C, ^31^P, ^17^F, etc.) and 2D experiments, such as COSY, NOESY, ROESY, TOCSY, DOSY, HETCOR, J–RES, INADEQUATE, HSQC, HMBC, etc., for liquids; and CRAMPS, CP/MAS, TOSS, DRAWS, REDOR, etc., for solids, considerably facilitate the identification of interactions occurring between NMs and surface ligands [80,81]. Thus, it has been shown that DOSY (diffusion ordered spectroscopy) makes the accurate assignment of specific resonances in 1D ^1^H spectra relative to bound ligands possible [82,83], while NOESY (nuclear Overhauser effect spectroscopy) enabled NM-ligand interactions to be pinpointed, even with ligands in a fast adsorption/desorption equilibrium and in complex solvent mixtures [84].

The possibilities offered by NMR to analyse bound ligands have been used by various research groups to study the composition of the ligand shell [85,86,87,88], to analyse the interactions between ligands and NMs [86,87,88,89,90,91,92,93,94,95,96] and to determine the relative binding strength of different ligands [97]. Moreover, it is being increasingly used to support studies that link physicochemical properties of NMs to the NM’s surface chemistry [95,98,99,100]. It has been reported that ^1^H and ^13^C signals in the QDs spectra shifted their position in comparison with the free ligand signals to lower field (higher frequencies), as a result of the proximity and anisotropic environment of the QDs surface [101].

**Figure 4:**
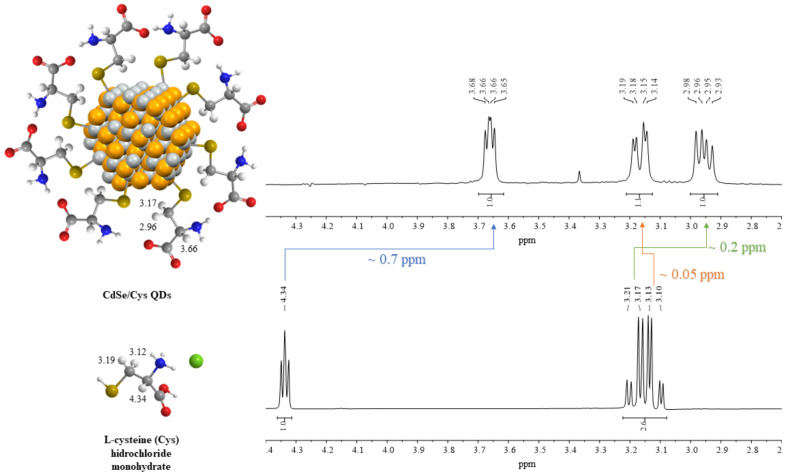
^1^H NMR spectrum (in D_2_O) of cysteine-capped CdSe QDs (**top**). The spectrum of L-cysteine (**bottom**) has been included for comparison.

We have characterised cysteine-capped CdSe QDs by ^1^H NMR spectroscopy in D_2_O (Figure 4). The analysis of the spectrum clearly shows the characteristic signals of methanetriyl (centred at 3.66 ppm) and methylene protons (at 3.17 and 2.96 ppm). The interaction between CdSe and anionic cysteine results in significant shifts of their signals. Thus, methanetriyl proton shifts 0.7 ppm towards high fields. The same is true for one of the methylene protons, although to a lesser extent (0.2 ppm). The remaining methylene proton moves in the opposite direction at only 0.05 ppm.

Moreover, NMR techniques allowed the quantification of ligands adsorbed to QDs by analysing those signals originated by protons of the ligands interacting with the QD surface by comparison with an internal standard [102]. DOSY offers the valued possibility to prove the attachment of surface ligands to NMs surface [103]. Additionally, a combined ^1^H–NMR/DOSY procedure can be a complementary method for the measurement of the NM’s size (hydrodynamic diameter, dH) and particle size distribution.

#### 3.2.2. Fourier Transform-Infrared Spectrophotometry (FT–IR)

The FT–IR technique has been successfully applied to the study surface effects on NMs, as it can provide a deep insight into the functional groups on the surface of functionalised NMs. It has been also employed to study the physisorption of molecules at the surface of NMs. Furthermore, the IR spectra of suspensions were used to extract information about specific surface area, surface charge density and concentrations of a nanomaterial.

Among others, carboxyl, hydroxyl, thiol or amine groups are the typical functional groups on the surface of functionalised NMs. In order to facilitate the study of the interaction between NM and organic ligands, the most characteristic stretching and bending vibrations of carboxyl, hydroxyl, thiol and amine functional groups are commented below. Changes affecting the wavenumber and intensity of their related IR bands can allow diagnosing what functional groups of the surface ligands might interact with NMs.

Non-coordinated organic ligands containing a carboxyl functional group show a very broad band for the O–H stretch in the region 3300–2500 cm^−1^, which is superimposed on the sharp C–H stretching bands of both alkyl and aromatic groups. The broadness of the O–H stretch band of carboxylic acids is related to the existing hydrogen-bonded dimers, which are usually formed by carboxylic acids. The O–H bending band is present in the regions 950–910 cm^−1^ and 1440–1395 cm^−1^, although the 1440–1395 band may not be distinguishable from C–H bending bands in the same region [104]. While the C–O stretch of a carboxylic acid appears in the region 1320–1210 cm^−1^, the C=O stretch appears as an intense band in the region 1760–1690 cm^−1^. The exact position of this strong band depends on whether the carboxylic acid is saturated or unsaturated, dimerised or displaying internal hydrogen bonding. Oluwafemi et al. [105] reported that bands at 1754 and 1294 cm^−1^, which correspond to the COO^−^ asymmetric and symmetric stretch of cysteine, shifted to lower wave numbers in CdSe/cysteine QDs. The shift in the COO^−^ stretching position is probably due to a change in the dipole moment, when cysteine is interacting with a metal surface with high electron density.

Furthermore, organic ligands having a hydroxyl functional group, such as alcohols and phenols, show a relatively broad and strong band for the O–H stretch in the region of 3550–3200 cm^−1^, which is characteristic of hydrogen-bonded hydroxyl groups. Besides the O–H stretching vibrations, there is a bending O–H vibration normally observed in the region of 1410–1260 cm^−1^. There also is a strong C–O stretching vibration between 1210 cm^−1^ and 1050 cm^−1^ [106]. Li et al. [107] reported that the characteristic broad band at 3399 cm^−1^ corresponding to the O–H stretch shown by carbon dots shifted to lower wave numbers (3306 cm^−1^) in C QDs decorated with a catechol derivative.

**Figure 5:**
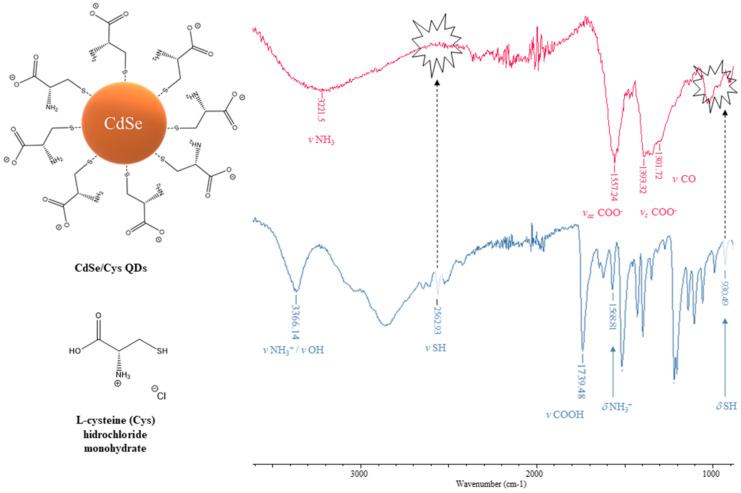
ATR–FTIR spectrum cysteine-capped CdSe QDs (**top**). The spectrum of L-cysteine (**bottom**) has been included for comparison purposes.

With regard to S–H and C–S stretches, thiols exhibit weak infrared bands (strong in Raman), which occur in the regions of 2600–2540 and 710–570 cm^−1^, respectively. In addition, the S–H bending vibration is normally observed at about 940 cm^−1^ [108]. The free surface ligands had an absorption band around 2500 cm^−1^ due to the S–H group. This band disappears in the case of capped QDs, indicating that the surface ligands are attached to the surface of CdSe QDs via their S atoms. Oluwafemi et al. [105] reported that the spectra of cysteine-capped CdSe particles lack bands at 2546 and 950 cm^−1^, which correspond to the S–H stretching and bending modes, respectively, in the free cysteine spectrum. This provides strong evidence for the cleavage of the S–H bond, and the subsequent formation of new S–Cd bonds of the Cd thiolate complex on the QD’s surface. For the purposes of illustration, the ATR–FTIR spectrum of cysteine-capped CdSe QDs prepared by us is shown in Figure 5, where the most characteristic infrared bands have been highlighted. This figure clearly shows the absence of νSH and δSH in the ATR–FTIR spectrum of cysteine-capped CdSe QDs, which indicates that the interaction occurred through the thiol group of the cysteine and Cd^2+^ ions on the surface of QDs.

N–H stretches of amines are typically in the 3300–3000 cm^−1^ region. These bands are weaker and sharper than those of the alcohol O–H stretches that appear in the same region. For primary amines (–NH_2_), there are two bands in this region, the asymmetrical N–H stretch and the symmetrical N–H stretch in the regions of 3400–3300 and 3330–3250 cm^−1^, respectively. Generally, secondary amines (–NH–) show only one band in the region 3350–3310 cm^−1^, while tertiary amines lack N–H bonds; consequently, they never show a band in this region. The N–H bending vibration of primary amines is observed in the region of 1650–1580 cm^−1^. A strong band attributed to N–H wag is observed only for primary and secondary amines in the region of 910–665 cm^−1^. The C–N stretching vibration of aliphatic amines is observed as medium or weak bands in the region of 1250–1020 cm^−1^. For aromatic amines, this band is usually stronger and appears in the region of 1335–1250 cm^−1^ [109]. Cooper et al. [110] investigated the characterisation of primary amine capped CdSe, ZnSe and ZnS QDs by using FT–IR, observing that octylamine-capped ZnS QDs display a broad band with two separate peaks at 3409 and 3281 cm^−1^. This means that the antisymmetric stretch was frequency upshifted by 39 cm^−1^, while the symmetric stretch was frequency downshifted by 13 cm^−1^ in comparison with that of free octylamine (3370 and 3294 cm^−1^). Likewise, the same authors have also observed a splitting of the –NH_2_ scissoring mode from 1610 cm^−1^ in the neat sample to 1584 and 1541 cm^−1^ (medium to strong bands) when interacting with ZnS QDs.

#### 3.2.3. Raman Spectroscopy

Raman spectroscopy, similarly to FTIR, affords information about intra- and inter-molecular vibrations, which can provide additional understanding about the interactions between QDs and surface ligands from a different point of view. Thus, IR spectroscopy relies on absorption of radiation, whereas Raman spectroscopy results from a light-scattering process, but it offers complementary information as, in many cases, molecular symmetry allows both Raman and IR activity. However, molecules containing an inversion centre, Raman bands and IR bands are mutually exclusive; i.e., a bond is either Raman or IR active, but not both. As a general rule, functional groups having significant changes in dipoles are strongly active for IR spectra, whereas functional groups showing weak dipole changes, or having a high degree of symmetry will be better observed with Raman techniques. Accordingly, bonds with strong dipole changes (e.g., C=O, O–H and N=O) and typically identified by IR are difficult to observe in Raman Spectroscopy. By contrast, many bonds, which are not so evident in FT–IR spectra (i.e., O–O, S–H, C=S, N=N, C=C etc.), can be more evident in Raman spectroscopy [108]. Therefore, Raman spectroscopy [111] is a better option than IR spectroscopy for investigating the interactions between QDs and surface thiol ligands, as with other NMs [112,113].

In recent years, Raman spectroscopy has been used to study a variety of QDs such as graphene QDs alone [114,115,116], CdS QDs-graphene hybrid material [117], Zn-containing QDs [118,119], GeSb QDs [120] and even Mn-doped CdSe QDs [121]. In these reports, Raman spectroscopy often is only one technique among different characterisation techniques (FTIR, XPS, TEM, etc.), but sometimes it is the only characterisation technique used [116,117,119].

#### 3.2.4. Spectrofluorimetry

Fluorescence spectrophotometry serves as a useful tool to understand structural changes at the NM-ligand interface by measuring the variation of fluorescence intensities [122,123,124,125,126,127,128,129,130]. Since fluorescence quenching or enhancement is strongly distance-dependent for NPs [131] and also for QDs [127,132], fluorescence spectrophotometry can provide information about the NM-ligand interface, which allows shedding light on the adsorption dynamics and mechanism.

The influence of pH or temperature on fluorescence [133,134] and the determination of the hydrodynamic radius of CdTe QDs [135] has also been reported.

#### 3.2.5. UV–Vis Spectrophotometry

This technique is particularly suitable for obtaining valuable information when organic ligands become bonded on NM surfaces because this binding may change the absorption spectra of surface ligands or NMs. In addition, these capping ligands provide the interface to many applications. Among the different ligands to be considered, long chain thiols, that some years ago appeared to be an excellent choice for most of semiconducting and metal NPs [136], have been revealed as one of the most successful cases of QD capping or functionalisation [137]. Yang et al. [137] cite thioglycolic acid (TGA), 3-mercaptopropionic acid (MPA), reduced glutathione (GSH), L-cysteine (L-Cys), sodium 2-mercaptoetahensulfonate (MES) and 1-thioglycerol (TG) as the most widely used thiol-capping agents. However, they only studied the complexation properties of MPA, GSH, TG and MES with CdTe QDs, combining theoretical calculations with experimental UV–Vis absorption spectra.

**Figure 6:**
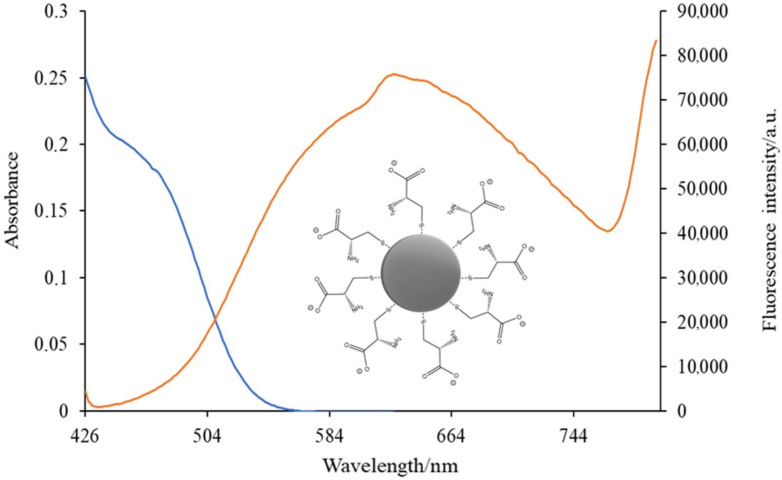
UV–Vis (**left**) and fluorescence (**right**) spectra of the cysteine-capped CdSe QDs in ethanol (λ_ex_ = 400 nm; λ_em_ = 632 nm).

Similarly, the association of empirical data (obtained from UV–Vis spectrophotometry and other characterisation techniques) with structural studies has been applied to investigate the gelation of GQDs (graphene QDs) [138] or the effect of size on optical properties of GQDs by using polycyclic aromatic hydrocarbons (PAHs) as molecular models [139]. Furthermore, the size of QDs can be determined from the position of the absorption peak in the UV–Vis spectrum [140] provided that a suitable model is used, as stated by Pesika et al. [141]. This offers a valuable support to prove the attachment of surface ligands to NMs surface. In this sense, the absorption wavelength (λabs) obtained by this technique, in joint connection with the equations proposed by Yu et al. [142], allows determining the size of CdS, CdSe and CdTe QDs in a simple and convenient manner. Thus, UV–Vis spectroscopy allowed us to observe a band around 460 nm, which led us to estimate the particle size in about 2.02 nm in the spectrum of cysteine-capped CdSe QDs (Figure 6). This also shows a broad fluorescence emission band at λ_max_ 626 nm, when an ethanol solution of cysteine-capped CdSe QDs was excited with visible light (400 nm).

A study on how size and shape influence on optical properties of oleic acid—as well as trioctylphosphine-capped CdSe QDs—has been reported too, using the results achieved by UV–Vis absorption, fluorescence spectra and X-ray diffraction [130].

Current models applied to relate the optical properties (of QDs or other NMs) and size distributions are often revised and discussed. Thus, an extensive revision by Ferreira et al. of the main theoretical contributions to the understanding of the quantum confinement effect and its consequences produced results comparable to those achieved by AFM and TEM data for CdTe QDs [143]. Their improvement consisted in including, in the model, the broadening of the absorbance spectrum caused by the polydispersity of the QDs, as it had been described some years before when working with ZnO QDs [144]. Later, Lesnichaya et al. [145] successfully applied this refinement of the method to Se NPs as confirmed by TEM data, thus supporting the applicability of the methodology to different systems of semiconductor NMs [143].

Closely related to the luminescence exhibited by QDs and photodetection technology, UV–Vis spectrophotometry is widely used, for example, for the characterisation of metallic Bi QDs [146] or Mn-doped CS QDs (ZnCdS:Mn/ZnS) [147].

#### 3.2.6. Isothermal Titration Calorimetry (ITC)

During titration, the heat change is directly recorded, and important thermodynamic parameters (including changed of enthalpy ΔH, entropy ΔS and Gibbs-free energy ΔG) and reaction parameters between QDs and surface ligands (including the binding constant and binding stoichiometry) can be calculated. Meanwhile, it also provides information on the interaction mechanisms between surface ligands and NMs. Although ITC is a straightforward technique for measuring the association process between surface ligands and NMs in a quantitative manner, its main inconveniences are that it is time-consuming and relatively high concentrations of titrant are needed.

ITC was firstly used to characterise the binding of QDs with human serum albumin (GSH) by Yang et al. [148], who also carried out spectrofluorimetric and UV–Vis spectrophotometric measurements. The ITC results revealed that this binding was a thermodynamically spontaneous process mainly driven by hydrophobic interactions, and the binding constant tended to increase as the GSH–CdTe QDs’ size increased. In this case, the binding constant decreased from 4.50 × 10^5^ to 1.34 × 10^5^ M^−1^ as the size decreased from 3.0 nm to 2.8 nm. Meanwhile, the binding stoichiometry for QDs (3.0 nm): GSH and QDs (2.8 nm): GSH at 298 K was determined to be 0.269 and 0.204, respectively.

Later on, the same authors carried out an analogous work to explore the effects of *N*-acetyl-L-cysteine-capped CdTe QDs on bovine serum albumin (BSA) and bovine hemoglobin (BHb) in vitro under physiological conditions [149]. Although proteins interacted spontaneously with the CdTe QDs, such interactions were deeper on BHb than on BSA, whereas the ITC results indicate that both binding processes are endothermic, and the predominant force is a hydrophobic interaction.

Based on previous theoretical studies showing that ligands derived from alkylphosphonic acids were more strongly bonded to CdSe QDs than carboxyl groups-containing ligands, Gee et al. [150] used ITC to monitor the carboxylate to phosphonate ligand exchange reaction on CdSe QDs in tetrahydrofuran (THF) at room temperature. The results reported confirm the potential and interest of ITC as another technique in the field of NM research.

#### 3.2.7. Size Exclusion Chromatography (SEC)

SEC is a column chromatographic technique that separates molecules mainly on the base of their relative size or, more specifically, on their hydrodynamic volume, and NMs can be separated likewise [151]. When samples are injected into the column, smaller molecules can be trapped into the pores of the gels, while larger molecules are excluded and firstly eluted. Since bare NMs have lower volume than capped NMs, the size distribution of the target NM after sorption can be determined, indicating that adsorption has occurred. Different detectors, such as SLS (static light scattering), QELS (quasi-elastic light scattering) and ICP–MS (inductively coupled plasma mass spectrometry), can be used in conjunction with SEC to generate size information. Although the use of the SEC as a separation technique is simple and fast, it is not possible to obtain information on the adsorption mode.

Recently, Wu et al. [152] described the use of this technique to purify CdSe/ZnS QDs modified with octylamine grafted polyacrylic acid (OPA) or with amino-terminated polyethylene glycol (PEG) to generate water-dispersible QDs (OPA–QDs and PEG–QDs). Since the objective was to prepare QD-based bioprobes, the authors took advantage of the separation capability of SEC to purify both the modified QDs and QD-based bioprobes, removing an excess of reagents and other substances. Three detection systems were used: UV–Vis, fluorescence and refractive-index detectors.

#### 3.2.8. Zeta Potential Determination

The adsorption of organic ligands on NMs can lead to a change in their surface charge, even so drastically as to reverse it, with the consequent influence on the zeta (ζ) potential. Thus, it was found that QDs carrying a PEG-amine had a ζ potential of −37.92 ± 0.16 mV (similar to the −31 mV reported for PEG–CdSe/ZnS QDs [152]), but when these QDs were conjugated to somatostatin (SST) to produce QDs–SST, the ζ potential showed a reversal to positive values (+23.3 ± 0.23 mV) [153]. More recently, water-soluble CdSe/ZnS QDs were encapsulated with modified poly(maleic anhydride-alt-1-tetradecene) (PMAT). PMAT was modified by using carbodiimide reactions between carboxyl groups of PMAT and primary amino groups of bifunctional molecules containing differently charged groups (sulphonic acid, phosphonic acid, trimethylammonium chloride, sulphate or phosphate) [154]. These modifications resulted in controlled ζ potential values ranging from −38 to +20 mV in an aqueous solution, while preserving the hydrodynamic diameter of the resulting QDs below 50 nm.

Furthermore, as the zeta potential depends on the type of charged groups, ζ potential analysis can be used to study the NM-surface ligand interaction either by physical or chemical adsorption. The number of organic ligands covalently attached to the surface of CdTe QDs has been also determined from their electrophoretic mobilities [155]. Although the ζ potential analysis cannot provide direct information on the surface binding, it can be useful as an important auxiliary technique to elucidate the adsorption mechanism.

In addition, the monitoring of the ζ potential with time allowed Wang et al. [156] to study the charge, size, aggregation behaviour and stability of 1-thioglycerol (TG)-CdTe QDs at different pH values, aiming at future applications in biosystems.

Another topic to highlight the interest of ζ potential determinations is the study of the dispersion of QDs in liquid crystalline materials (or liquid crystals, LCs). LCs exhibit interesting dielectric, optical and spectroscopic properties that explain their wide variety of applications. Among the different phases in LCs, the nematic phase is the simplest one and has very interesting advantages in the fields of both display and non-display electronic devices [157]. In addition, the dispersion of NMs, namely QDs, in nematic LCs (NLCs) has gained importance due to the improvement in the properties that QDs dispersion impairs NLCs [158]. Likewise, in the previous paragraph, the study of the stability and aggregation of pristine and QD-dispersed NLCs was carried out by ζ potential and spectrometric (UV–Vis and fluorescence) measurements, when dispersing CQDs in 4′-heptyl-4-biphenylcarbonitrile [158], and when oil palm leaf-based carbonaceous QDs (OPL QDs) were dispersed in the commercial nematic mixture E48, a positive (+ve) dielectric anisotropic material with the phase sequence Cr.–19 °C–N–100 °C–Iso was observed [157].

#### 3.2.9. Powder X-ray Diffraction (XRD)

Powder X-ray diffraction (XRD) is a rapid analytical technique that has been primarily used for the phase identification of a crystalline material, but it is a common characterisation technique for finely ground and homogenised nanoscale materials as well. It can provide information not only about unit cell dimensions but also about average bulk composition, purity, phase identification, size, crystallinity and, in some cases, morphology and other important features of nanoscale materials. However, unambiguous sample characterisation almost always requires complementary experimental microscopic and/or spectroscopic methods and/or computational methods [130,159]. Thus, as a bulk technique, the information that is provided can be correlated with microscopy data to test if microscopic observations on a small number of particles are representative of most of the sample. X-ray diffraction (XRD) patterns for samples of nanoparticles possessing different sizes and shapes can look different, and a careful analysis of the XRD data can provide useful information and help correlate microscopic observations with the bulk sample.

Additionally, a deep study of XRD spectra, their comparison with simulated X-ray diffraction patterns and the results achieved with other techniques can help explain the origin of the ligand signals recorded in such spectra, which can be considered as a probe of ligand-shell ordering, as described by Calvin et al. in the case of InP QDs [160].

More detailed XRD spectra can be obtained, if necessary, by high resolution XRD (HR–XRD). Thus, HR–XRD and photoluminescence have been used to study active media for lasers and light emitting diodes. See, for example, the following cases: the study of GaAs/In_0.15_Ga_0.85_As/GaAs quantum wells (QWs) with embedded InAs QDs [161], the characterisation of GaAs/Al_0.30_Ga_0.70_As QWs with InAs QDs arrays covered by different capping layers (Al_0.30_Ga_0.70_As or Al_0.1_Ga_0.75_In_0.15_As) [162], and the investigation on the effect of the same capping layers on the emission of GaAs/Al_0.30_Ga_0.70_As/AlGaInAs heterostructures with embedded InAs QDs [163].

#### 3.2.10. X-ray Absorption Spectroscopy (XAS)

Extended X-ray Absorption Fine Structure (EXAFS) and X-ray Absorption Near Edge Structure (XANES) are subsets of X-ray absorption spectroscopy (XAS). X-ray absorption spectroscopy (XAS) is one of the direct structural probes providing information on the local environment around a photoabsorber. XAS is an excellent tool for this purpose, because it can be successfully applied to both ordered and disordered materials. XAS is also element selective, and it is sensitive to high dilutions and to length scales down to NMs and even molecules. X-ray absorption spectroscopy is well suited for the investigation of nanomaterials, and size-dependent effects are readily detectable for nanoparticles having a size below about 10–15 nm. An analysis of the nearest coordination shells around a photoabsorber can be performed using conventional methods of analysis to determine the local structure parameters and degree of disorder. The combination of XANES and EXAFS analyses has proved to be a robust methodology for the characterisation of complex systems [164,165], even when only EXAFS measurements are obtained and XANES spectra are used only for comparison purposes [166,167]. This approach can provide both local symmetry information (through XANES), as well as structural and stoichiometry information (through EXAFS), which can be used as an input for the subsequent analysis of scattering data and computer modelling [165] or as a support to density functional theoretical (DFT) calculations [168]. However, EXAFS or XAS are often used alone. Thus, EXAFS has been used to investigate the microstructure of multilayer semiconductor heterosystems SiGe containing self-assembled ordered groups of Ge QDs [169], while XAS has been used to study the electronic structure and the electrochemical performance of NiCo_2_O_4_/graphene QDs [170].

#### 3.2.11. X-ray Photoelectron Spectroscopy (XPS)

As described above, because of their small size, QDs have a high surface-to-volume ratio; thus, many of their properties are surface chemistry-dependent [171]. Hence, surface analysis techniques are of high interest, and X-ray photoelectron spectroscopy (XPS) is deserving of particular mention. In fact, the literature shows that XPS has been the technique of choice for the analysis of the surface of a variety of QDs [8,171,172,173,174,175,176,177], although as previously mentioned, authors often combine XPS and other techniques with characterization purposes.

N-doped GQDs synthesised by a top-down electrochemical exfoliation process were also studied by XPS prior to being used in the fabrication of a novel GQDs-based light-emitting electrochemical cell (LEC) [8]. CdSe QDs were also subjected to XPS to study the desorption dynamics of different ligands upon annealing treatments at different temperatures [172]. In the case of He et al., they have used XPS to characterise N-doped GQDs prepared through a hydrothermal process and achieved the complete elucidation of the full band structures of such QDs [173]. Therefore, the complete elucidation of the structure of core-shell QDs is indeed possible, as also occurring in Weigert et al.’s study with CdSe/CdS QDs [177], showing the potential of complementary techniques such as HR-TEM and XPS. These same techniques allowed the study of the growth and the effect of post-annealing process on the crystallinity and crystallite size of SnO_2_ QDs [174].

The Mn-doping of CdSe QDs was also investigated by XPS, since such doping was predicted to optimise the surface passivation of anionic and cationic surface trap states, which organic ligands cannot overcome [175]. Moreover, the use of passivation strategies on QDs is well-known, since without a well-passivated surface, QDs are prone to oxidation, resulting in poor performance and shorter lifetime. Thus, XPS on depth-profiling modes can be useful for studying the variation of the extent of oxidation processes with depth, as reported by Clark and Flawell [171], who have also investigated PbS/CdS QDs by XPS.

Finally, XPS is not only useful for investigating “simple” QDs but also much more complex structures, such as the 6-fold stack of Si QDs layers embedded in an ultrathin SiO_2_ matrix. This material was prepared by Futamura et al. [176], who investigated the electron emission mechanism when the structure was subjected to DC biases, concluding that electrons gain kinetic energy mainly in the upper side of the stacked Si QDs structure.

#### 3.2.12. X-ray Fluorescence (XRF)

XRF is a non-destructive analytical technique used to determine the elemental composition of materials. X-ray fluorescence (XRF) results from the atom-localised emission when incident X-rays or γ–ray with a higher energy than the ionisation potential of the atoms in the NMs induces the emission of characteristic fluorescence [178,179].

Besides elemental compositions, XRF can help find the precise position of functional NMs inside three-dimensional structures with photonic or other properties, as described by Schulz et al. [180]. They infiltrated PbS QDs deep inside crystals made of Si and localised them using synchrotron XRF tomography.

Another application of XRF as a characterisation technique deals with the development of contrast agents to be adopted in X-ray computed tomography: silica-coated Au nanospheres (Au@SiO2) were prepared and coated first with CdSe/ZnS QDs and then coated again with silica and finally modified with polyvinylpyrrolidone (PVP). The composite Au@SiO_2_–QDs/SiO_2_–PVP structure obtained was first studied by XRF and other techniques, revealing good dispersity, high fluorescence intensity and good effects of X-ray absorption [181]. The subsequent in vivo application of the probe on mice was reported to be successful, and it is expected to be used for tumour-targeted and vascular imaging [182].

#### 3.2.13. Scanning Electron Microscopy with Energy Dispersive X-ray Analysis (SEM–EDX)

**Figure 7:**
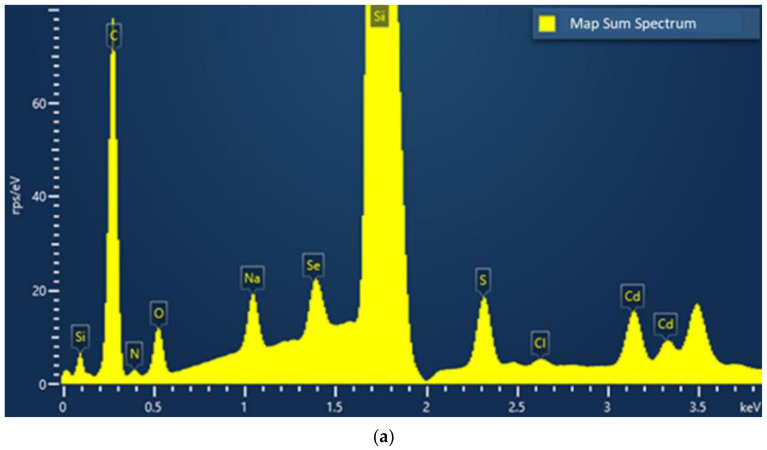

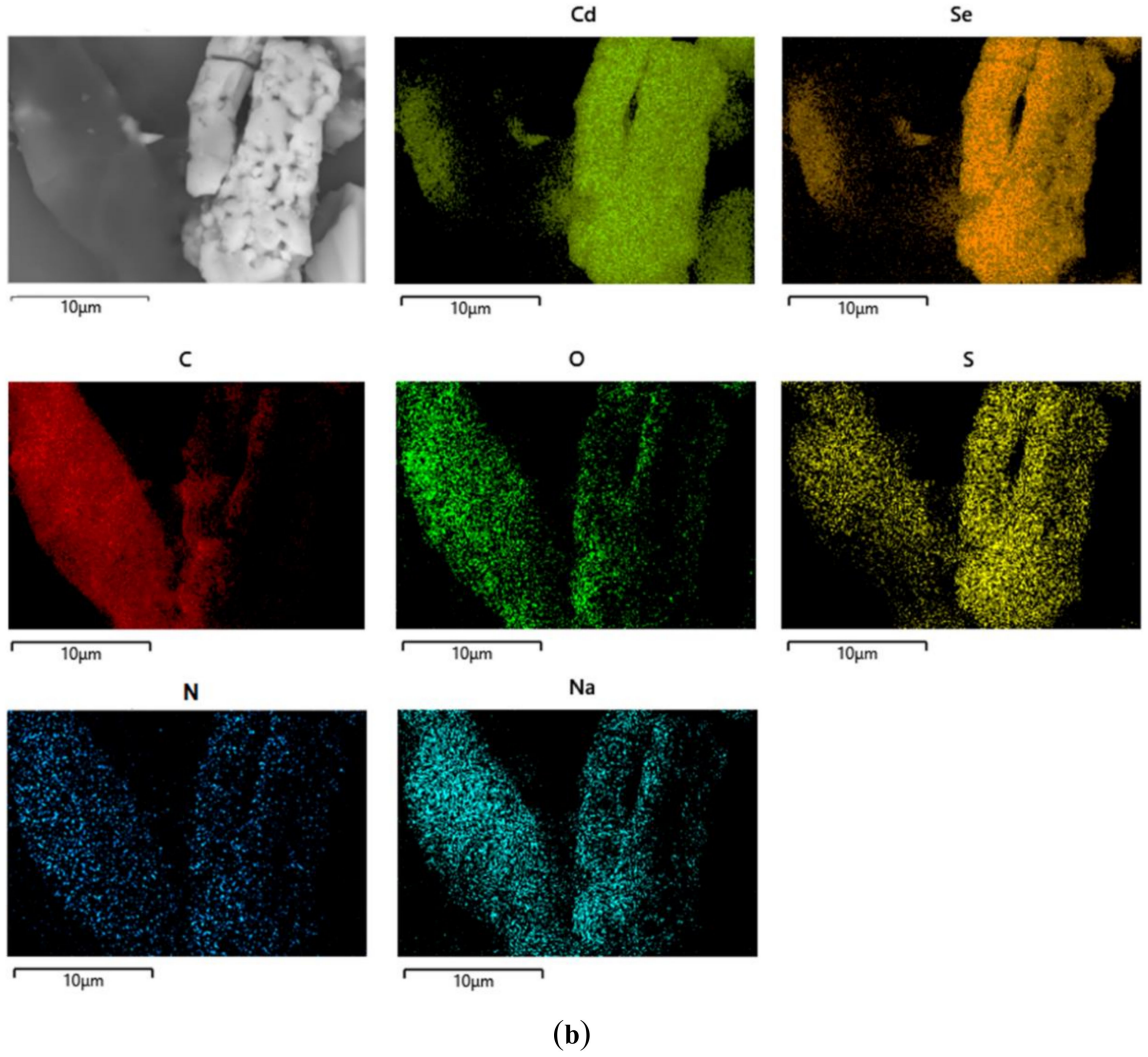
(**a**) EDX spectrum of CdSe–Cys QDs; (**b**): SEM micrograph with EDX mapping pattern of the elements Cd, Se, C, S, N, Na and O from an anionic cysteine-capped CdSe QDs sample.

SEM provides detailed, high-resolution images of the nanomaterial by scanning a focused electron beam across the surface and detecting the secondary or backscattered electron signal. This often allows the size and shape of nanomaterial particles to be studied [183,184]. An EDX analyser is used to provide elemental identification and not quantitative compositional information (% weight of the constituent in the nanocrystal) [185] as well as element distribution in nanomaterials (mapping analysis) [186,187]. The sample is irradiated with electrons resulting in the emission of X-rays characteristic to the elements present. The energy emissions are translated into spectral peaks of varying intensity, resulting in a spectrum profile, which identifies the different inorganic elements present in the sample. The size of spectrum peaks is directly proportional to the concentration of the elements in the sample. Figure 7a shows the EDX spectrum of cysteine-capped CdSe QDs. The huge size of the silicon peak is due to the sample holder, which contains this element.

SEM with EDX has been used to generate a map of element distributions from cysteine-capped CdSe QDs (Figure 7b). It must be noted that cysteine, as a carboxylate sodium salt, consists of O, Na, C, N and S elements. The EDX analysis shows the presence of Cd, Se, O, Na, C, N and S elements with a very similar and uniform spatial distribution pattern, which supports the existence of the interaction of anionic cysteine with CdSe.

## 4. Analytical Techniques for Detection and Quantification of QDs

While the characterisation of synthesised QDs is widely covered in the literature, the research on the detection or quantification of QDs in complex samples is much more unusual. Thus, the detection/determination of QDs both directly (as inorganic or metal-free materials) [7,188,189,190,191,192,193] and indirectly (as the heavy metal content of inorganic materials) has been reported [11,12,54,56,194,195,196,197,198,199,200,201,202,203,204,205,206,207,208,209,210]. Nevertheless, for the indirect determination to be successful, the nature of the sample must be ensured; otherwise, the heavy metal content could come from sources other than the QDs themselves.

**Figure 8:**
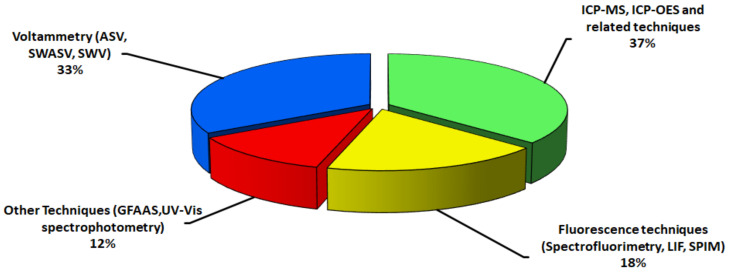
Sector graph of analytical techniques for detection/determination of QDs.

As depicted in Figure 8, voltammetric-sensing techniques [12,54,194,200,201,203,205,206,207,208,210] and mass spectrometry-related techniques [12,56,191,192,193,195,196,197,199,202,204,209] are commonly used analytical techniques for the detection/determination of QDs. Less used techniques are spectrofluorimetry [7,188,189,190,211,212] and others such as UV–Vis spectrophotometry or graphite furnace atomic absorption spectrometry (GFAAS) [142,194,213,214].

### 4.1. Electroanalytical Techniques: Voltammetry

Voltammetry is the general term for all techniques in which the current is measured as a function of electrode potential. Voltammetric-sensing techniques are characterised by simplicity, high sensitivity, good stability, low-cost instrumentation and small sample requirements. To our knowledge, the recently published literature on the detection of QDs reports the use of Square Wave Anodic Stripping Voltammetry (SWASV), Square Wave Voltammetry (SWV) and Anodic Stripping Voltammetry (ASV) techniques.

#### 4.1.1. Square Wave Anodic Stripping Voltammetry (SWASV)

A reliable and low-cost three-electrode microchip, produced by screen-printing technology, was used for the SWASV detection of CdS QDs, such as lab-made solutions [12]. CdS QDs are adsorbed on the working electrode, where Cd^2+^ ions are reduced to Cd^0^. The subsequent oxidation of Cd^0^ to Cd^2+^ allows its electrochemical detection in NaCl 0.05 M solutions (acetate buffer solution pH 4.6). The authors report a linear range in the graph with concentrations of CdS QDs between 50 and 8000 ng mL^−1^, a relative standard deviation (RSD) of 6.5% and a sensitivity of 0.0009 μA/(ng mL^−1^). The proposed sensor allows the use of much lower sample volumes than other devices, which opens the way to a variety of applications in microreactions, separations or pre-concentrations.

More recently, Sýs et al. proposed an SWASV method to determine several heavy metals likely have originated from the dissolution of QDs, which suggests the possible indirect determination of QDs [207]. This possibility became true shortly afterwards, when the detection of CdSe/ZnS QDs and PbS QDs by the same technique was published [208]. After studying the conditions for the determination of each type of QDs separately (linearities ranged from 2.5 to 15 nM CdSe/ZnS QDs and from 0.5 to 10 nM PbS QDs), the simultaneous detection of both QDs without any signal overlaps was achieved. The application of these findings to the determination of biomolecules, using QDs as sensitive tags bonded to them, seems to be at hand in the near future.

#### 4.1.2. Square Wave Voltammetry (SWV)

Square Wave Voltammetry has been reported as an inexpensive procedure for the rapid screening, field analysis and development of electrochemical biosensors. The SWV detection of CdS–GSH QDs was performed at pH values of 3.0 and 7.0 [54]. Different screen-printed electrodes produced reproducible signals (RSD 6.7%), although better responses were achieved by using one electrode for each measurement. Calibration was possible over the range 0.5–14.0 × 10^16^ QDs mL^−1^ (detection limit of ca. 2 × 10^14^ QDs).

The SWV detection of CdSe/ZnS–biotin QDs in an ammonia solution has been reported [205]. The authors report a linear range in graph with concentrations of CdSe/ZnS–biotin QDs between 0.05 and 2 nM (detection limit of 37 pM).

#### 4.1.3. Anodic Stripping Voltammetry (ASV)

The ASV detection of CdSe/ZnS-biotin QDs in an ammonia solution has been reported [203]. The method takes advantage of the catalytic effect of the QDs on the electrodeposition of Ag at the surface of QDs. The final stripping of the electrodeposited Ag was carried out by differential-pulse voltammetry. The direct relationship between the voltammetric signal and the concentration of CdSe/ZnS-biotin QDs allows its determination.

The ASV detection of CdSe/ZnS QDs and CdSe QDs in organic medium in water after derivatisation with an amphiphilic polymer has been also reported [201]. The detection limits achieved with the selected experimental conditions were 3.0 × 10^12^ nanoparticles mL^−1^ for CdSe QDs dispersed in organic medium and 6.0 × 10^12^ nanoparticles mL^−1^ for water-solubilised CdSe/ZnS QDs. The method can be applied to other QDs, and since the results are expressed in number of QDs per unit volume, the procedure can be useful for the study of toxicological and bioanalytical uses and risks of QDs.

A very sensitive method was applied to the ASV determination of CdSe/ZnS-streptavidin QDs and CdSe/ZnS-biotin QDs [206]. The use of a magnetoelectrochemical supports for screen-printed electrodes to improve the anodic stripping voltammetry of cadmium due to the generated magnetohydrodynamic (MHD) effect has been reported. To create a significant MHD effect, Fe^3+^ was added at mM concentrations to the solution. The reduction in iron(III) simultaneously with the cadmium deposition on the electrode surface allowed the production of a high cathodic current, which generated a large Lorentz force capable of exerting a convective effect on the solution in the presence of the magnetic field. The authors report a linear range in the graph with concentrations of CdSe/ZnS-biotin QDs between 0.05 and 5 nM (detection limit of 0.05 nM).

The detection of organic-capped CdSe QDs via cadmium deposition and ASV measurement was achieved down to the highly dilute concentration of 15 pM [200]. It has been reported that the use of a large capping agent alters the mass transport and solubility of an electroactive species adjacent to the electrode. The altered mass transport regime adjacent to the electrochemical interface can be utilized to afford a highly sensitive analytical signal for the detection of CdSe QDs.

The determination of the number of Zn and Cd atoms in CdSe/ZnS QDs was achieved from ASV measurements [194]. The comparison of the results obtained from ASV and atomic absorption spectrometry (GFAAS) showed good coincidence. In contrast, a comparison of the results with concentrations obtained by UV–vis spectrophotometry revealed large discrepancies. The authors attribute this to the different nanocrystals’ absorption cross sections from different synthesis routes. ASV has been also applied for the determination of novel Ag_2_S QDs with two different surface coatings: 3–mercaptopropionic acid (MPA) and boronic acid (BA) [210]. The LODs achieved were 4.10 × 10^10^ QDs mL^−1^ for MPA–Ag_2_S QDs and 5.70 × 10^10^ QDs mL^−1^ for BA–Ag_2_S QDs, both with good precision and with a wide linear response range (10^9^ to 10^12^ QD mL^−1^).

### 4.2. Atomic Spectrometry Techniques

#### 4.2.1. Inductively Coupled Plasma-Mass Spectrometry (ICP–MS)

ICP–MS is a technique useful to determine low concentrations (µg L^−1^) and ultra-low concentrations of elements (ng L^−1^). Atomic elements are led through a plasma source where they become ionised. Then, these ions are sorted on account of their masses. The advantages of the ICP–MS technique over AAS (Atomic Absorption Spectrometry) or ICP–OES (inductively coupled plasma optical emission spectrometry) are lower detection limits, larger linear ranges and possibilities for detecting the isotope composition of elements. The ICP–MS technique has a multi-element character and a high sample throughput (similarly to ICP–OES), but it allows the performance of more sensitive measurements. The disadvantages and weaknesses of the ICP–MS are the occurrence of spectral and non-spectral interferences and the high costs.

ICP–MS can identify the nature and/or concentration of dissolved ions released into water during QD-polymer composites degradation. When combined with centrifugal filtration separation techniques, ICP–MS can also quantify the ratio of released nanoparticles to released ions. Single-particle ICP–MS (sp–ICP–MS) can discriminate and quantify ions and nanoparticles. However, sp–ICP–MS can only discern the presence of nanoparticles with an element-dependent size limitation of 11–20 nm for cadmium, 21–80 nm for zinc and > 200 nm for selenium.

The Cd concentration from a small planktonic crustacean (*Daphnia magna*) exposed to of amphiphilic polymer coated CdSe/ZnS QDs was determined by ICP–MS [11]. Fluorescence confocal laser scanning microscopy was used to visualise and spectrally distinguish QDs from competing autofluorescent signals arising from the daphnia themselves and their food sources. Fluorescence emission help localise QDs within organisms and to assess their elimination and accumulation in the digestive tract.

Since the toxic effects of QDs are mainly ascribed to the release of ions derived from their chemical components and to the generation of reactive oxygen species (ROS) [56,192,209], the determination of the elements likely to be released from QDs has been carried out from different points of view. A suspension of polyethylene glycol-coated CdSe/CdS QDs (PEG–CdSe/CdS QDs) was applied locally to the skin of mice (in some individuals after the removal of epidermis), and Cd was determined by ICP–MS in sentinel organs (liver tissue and lymph nodes) [195]. The Cd levels found showed that damaged skin enabled the penetration of QDs, which is toxicologically relevant and enables the determination of QDs, as stated by Sewell et al. [197]. These authors determined QDs by means of ICP–MS determination of Cd and Se from streptavidin-functionalized CdSe/ZnS QDs. They found that Cd and Se were independent of the QDs functionalisation, and the QDs concentrations were not different from those obtained by UV–Vis spectrophotometry. Another approach to the study of citotoxicity of CdSe QDs [209] was the incubation of human hepatocellular carcinoma cells (HepG2) for 24 h with CdSe QDs, followed by magnetic solid phase microextraction (MSPME) and a subsequent ICP–MS sensitive determination of Cd and Se (with LODs of 2.2 and 21 ng L^−1^, respectively).

Given the described toxic effect of the elements released, other authors forced that process. The concentration of Cd released into solution as a result of an artificially accelerated photodegradation of CdSe/ZnS and CdSe QDs polymer composites was measured using ICP–MS [56], and cadmium-containing species, <0.45 µm in size, were detected with ICP–MS.

The single-cell ICP–MS (SC–ICP–MS) method was established to determine intracellular carboxyl-coated CdSeS QDs in single cells after exposure. The results were compared and validated by flow cytometry and cell digestion methods. In contrast to other methods, SC–ICP–MS can directly detect QDs and their degradation products via elements [199]. Cells are sprayed in sequence into a high temperature plasma, where each cell is desolvated, and its constituents are atomised and ionised. The resulting ions are then detected by mass spectrometry. In the mass spectra, the intensity of each transient signal corresponds to atomic constituents in a single cell, and the frequency of transient signals is directly related to the number of cells. The number of cells detected by ICP–MS (F_cd_) during acquisition time (t) can be calculated by the following equation:

$$F_{\mathrm{cd}}=\varepsilon \cdot Q_{\mathrm{sam}}\cdot N_{\mathrm{cd}}\cdot t$$

where ε is the transport efficiency, Q_sam_ is the sample uptake rate (mL s^−1^), N_cd_ is the cell number density (mL^−1^), t is the acquisition time (s) and F_cd_ is the number of cells detected. It is necessary for SC–ICP–MS analysis that only one cell enters the plasma at a time so that each transient signal in the mass spectrum corresponds to a single cell.

In addition, the use of ICP–MS as a detector in hyphenated techniques has also been reported. Size-exclusion chromatography coupled with ICP–MS as a detector (SEC–ICP–MS) was used to separate and quantify CdSe/ZnS QDs and their dissolved metal cations (Cd^2+^ and Zn^2+^). This was made on lab-prepared suspensions and in spiked water samples (river water, moderately hard groundwater and a secondary effluent from a municipal wastewater treatment plant) [191]. The method enabled the determination of QDs at 1 µg L^−1^ or more, and the results agreed with the achieved with the conventional method involving previous separation of dissolved cations by centrifuge ultrafiltration. Moreover, regarding the release of cations from the QDs, the authors observed that the QDs leached ca. 50% Zn^2+^ and 20% Cd^2+^ over 4 days within natural river water contained within a 2 mL HPLC autosampler vial. Moreover, SEC–ICP–MS was the technique applied for the speciation of four CdSe/ZnS QDs in HepG2 cells: cells were incubated with QDs and later fractioned by SEC–ICP–MS [192]. The method was based on metallomics, a novel omics science, which was developed in recent years by focusing on the amount, speciation, distribution, structure and function of metals in biological systems. This is quite interesting, because little is known about the QD species present in media after their degradation. Eventually, two types of chemical form, named as QD–1 and QD–2, were found in HepG2 cells. QD–1 and QD–2 were confirmed to be a type of QD-like nanoparticles and a type of Cd-metallothionein complex, respectively.

Asymmetric flow field-flow fractionation (AF4) coupled with ICP–MS is another hybrid technique to be mentioned. AF4–ICP–MS has been proposed for the detection of QDs in the monitoring of bioconjugation between a protein, namely a “model” monoclonal IgG antibody (Ab) and CdSe/ZnS QDs (which were surface-coated with an amphiphilic polymer) [202]. Cd, Se and S were determined, which is a critical need for the assessment of the elution of the bioconjugated or free Ab. The LOD obtained, as QD concentrations, was 11 ± 3 nM. Later, through the determination of Zn by AF4–ICP–MS, the concentration of Mn^2+^-doped ZnS QDs was estimated as the number of QDs per unit volume and number of surface ligands on the QDs (ligand density). These two parameters help in assessing the reliability of quantitative analytical or bioanalytical applications of the QDs [204]. L-cysteine and dihydrolipoic acid (DHLA) were compared as surface ligands, and the latter one was found to enhance the phosphorescent properties of capped QDs and their aqueous stability, while facilitates the further bioconjugation of the QDs to biomolecules.

Finally, capillary electrophoresis coupled to ICP–MS (CZE–ICP–MS) enabled the separation of L-glutathione/L-cysteine-capped CdTe QDs, and the corresponding ionic species, Cd^2+^ and TeO_3_^2−^, likely to be released from them [193]. The method was applied to buffered solutions and complex biological matrices (cell culture solution and normal rat serum sample). The interest of ions determination is their toxicity, as commented above [56,192,209]. All the analytes were completely separated, detected and quantified, with good sensitivity (LODs below 5.5 µg L^−1^), precision and analytical recovery.

#### 4.2.2. Inductively Coupled Plasma-Optical Emission Spectrometry (ICP–OES)

Inductively Coupled Plasma-Optical Emission Spectroscopy (ICP–OES) is a multi-elemental analysis technique capable of determining and quantifying, at concentrations ranging from % to ppb, most of the elements of the periodic table, with the exception of C, N, O, H, F, noble gases, some rare earths and other rare elements. The samples are introduced in liquid form, transformed by a nebuliser into an aerosol and excited by an argon plasma. The emissions of the excited atoms are collected by an optical system based on a polychromator combined with CCD detectors, obtaining an emission spectrum for the selected lines of each element.

The study of the extent of cadmium release upon exposure to a series of environmental and biological simulant fluids has been reported, in addition to the tracking of the loss of QD-characteristic fluorescence, as a marker for chemical damage to the CdSe/ZnS nanoparticles [198]. ICP–OES was used to distinguish soluble cadmium from particulate forms. Probably due to the lower sensitivity of ICP–OES compared to ICP–MS, ICP–OES is usually not the technique of choice.

#### 4.2.3. Graphite Furnace Atomic Absorption Spectrometry (GFAAS)

Although GFAAS is a powerful analytical technique for the analysis of elements present in complex samples such as biological and environmental samples by measuring the radiation absorbed by the target element, its use in the field of QDs determination is limited.

The determination of the Cd concentration by means of GFAAS was followed by the calculation of the amount of Cd atoms in each nanocrystal [194]. The CdSe QDs concentrations were determined by factoring the number of Cd atoms per nanocrystal of relevant size (AF) into the calculation. These values were calculated with the crystallographic software Diamond 3.0. The exponential fitting curve for the total amount of cadmium atoms (y) in one nanocrystal at a given emission wavelength (nm) is stated below.

$$y_{\mathrm{Cd}}=0.0952\cdot 10^{0.0168\;\lambda },{\;R}^{2}\;=\;0.9914$$

### 4.3. Molecular Spectrometry Techniques

#### 4.3.1. Spectrofluorimetry

Molecular Fluorescence Spectrophotometry has been used to study aqueous solutions containing graphene QDs (GQDs), which allowed its quantification [7]. GQDs are retained in a strong anion exchange column for their preconcentration, recovered in an aqueous solution and measured in a spectrofluorimeter using the fluorescence of the GQDs as the analytical signal for the quantification. The limits of detection and quantification were 7.5 mg L^−1^ and 25 mg L^−1^, respectively. The precision for 200 mg L^−1^, expressed as %RSD, was 2.8%. Recovery percentages between 86.9 and 103.9% were obtained for two different concentration levels. Interferences from other nanoparticles were studied, and no significant changes were observed at the concentration levels tested.

An approach based on single-molecule two-colour coincidence detection was developed to evaluate the on-state QDs in a microfluidic flow, taking advantage of the fact that single QDs exhibit the dynamic fluctuation of fluorescence intensity (i.e., blinking) with the transition between on and off states [211]. The authors have quantified the on-state QDs by detecting the coincidence signals of red streptavidin-coated CdSe/ZnS QDs associated with the green fluorescent microspheres in a microfluidic flow, which can overcome the limitations that are inherent in the above immobilisation method and fluorescence correlation spectroscopy methods.

Spectrofluorimetric detection has been combined with poly(acrylamide) gel electrophoresis (PAGE) for the characterisation of CdSe/CdS/ZnS QDs [212].

#### 4.3.2. Laser-Induced Fluorescence Spectrophotometry (LIF)

In LIF, the excitation step is produced by a laser source. The most important advantages of this technique are low background signal, high selectivity towards the analyte, the obtention of information about the rotational-vibrational structure of the ground or excited state of the sample, and time-resolved information if pulsed lasers are used. In addition, polarisation-dependent measurements are easy to implement since most laser beams are linearly polarised.

It has been reported the quantification of the 3.1 nm QDs–TOPO/TOP/SDS by using LIF detection (excitation 480 nm and emission 520 nm) [189]. The surfactants used to achieve the surface functionalisation were trioctylphosphine oxide/trioctylphosphine (TOPO/TOP) and sodium dodecyl sulfate (SDS). A log-linear regression was found to provide the following equation: Y = −0.21 + 0.215 log X, R^2^ = 0.979, where Y is the peak area, and X is the concentration of the QDs–TOPO/TOP/SDS complex. These free QDs were separated by capillary zone electrophoresis (CZE) based on the differences in the charge-to-mass ratio of the QDs–TOPO/TOP/SDS complexes, and the detection was carried out with UV–Vis and laser-induced fluorescence (LIF) techniques obtaining detection limits 5-times lower with CE–LIF.

#### 4.3.3. Selective Plane Illumination Microscopy (SPIM)

SPIM is a fluorescence microscopy technique that uses a focused light sheet to illuminate the sample from the side. SPIM achieves excellent resolutions at high-penetration depths while being minimally invasive at the same time. SPIM offers a number of advantages over established techniques such as strongly reduced photo-bleaching, high dynamic range and high acquisition speed. It has been reported a setup for SPIM, which enables the detection and tracking of single streptavidin-coated CdSe/ZnS QDs in model biological systems, with the resolution of confocal microscopy and the optical penetration beyond 300 μm [188,190].

#### 4.3.4. UV–Vis Spectrophotometry

The concentration determination was performed by taking the extinction coefficient into account [180]. The CdSe/ZnS QDs concentrations determined by Schmelz et al. [213] and Yu et al. [213] provided a much higher Cd concentration in the solution if compared to the ASV and AAS data. However, the extinction coefficient and, thus, the concentration determined by Striolo et al. [214] seem to be comparable to the ASV and AAS. Due to the large discrepancies from different work groups, it is conceivable that the extinction coefficient of nanocrystals in comparison to organic fluorophores is additionally a function of the crystal’s quality. High crystallinity, i.e., fewer defects, guarantees higher quantum yields. Since the quantum yield of nanocrystals is strongly influenced by surfactants, their coverage rate to nanocrystals will differ not only in terms of the photoluminescence characteristic but also, conceivably, in terms of the absorption cross section. This can lead to a different extinction coefficient. Hence, nanocrystals prepared by different syntheses should be compared carefully, even if they have similarly shaped absorption characteristics.

## 5. Concluding Remarks and Prospects

The composition of NMs determines the size from which a material exhibits quantum confinement effect and, therefore, the unique optical and electronic properties of QDs. Semiconductor materials of a size less than double the bulk exciton Bohr radius undergo quantum confinement. Since electrons within QDs are confined to discrete energy levels, the wavelengths associated to the formation and recombination of excitons, i.e., the UV–Vis absorption and fluorescence emission of QDs, respectively, are tuneable by changing the particle’s size. An appropriate selection of both size and composition can lead to fluorescence wavelengths in both visible (380–750 nm) and near-infrared (750–2500 nm) ranges. Type I CS QDs are the most common in bioanalytical applications since this configuration provides the best confinement of the exciton and the highest rates of radiative electron-hole recombination (i.e., brightness of the photoluminescence).

QDs are frequently coated with an external layer or protective shell responsible for stability, solubility and functionalisation. Organic ligands with hydrophilic tails, such as –COOH, –OH, –SH, etc., are chosen to produce water soluble QDs. By contrast, ligands featuring a hydrocarbon tail, such as trioctylphosphine/trioctylphosphine oxide (TOP/TOPO), long-chain alkylamines and alkylthiols, etc., can afford QDs with higher solubility in nonpolar solvents. The external layer of passivating ligands enhances the stability of QDs by preserving under-coordinated atoms existing on the outermost layer of the core material against chemical processes such as oxidation and degradation. This is of crucial importance in QDs composed of a cadmium-based core material, such as those used as diagnostic and therapeutic tools in nanomedicine.

Because of their small particle size, QDs have a high ratio of surface atoms to core atoms, which causes the surface atoms to play a decisive role not only in properties but also in the reactivity of the entire particle. The lattice termination on the QD’s surface has as a consequence that surface atoms are typically undercoordinated, which makes them more reactive than the core atoms. The typical processes in the reaction of dissolved ligands (adsorbates) with nanomaterial surfaces (adsorbents) can be classified into two general types: chemisorption and physisorption. Forces such as weak hydrogen bonds, electrostatic interactions, van der Waals interactions, hydrophobic interactions, etc., are involved in physisorption. The forces involved in chemisorption are valence forces of the same type as those operating in the formation of chemical compounds, such as the formation of coordinate bonds (surface complexation).

The possibilities offered by NMR to analyse bound ligands have been used by various research groups to study the composition of the ligand shell, to analyse the binding between ligands and NMs and to determine the relative binding strength of different ligands. NMR allowed the quantification of the ligands adsorbed to QDs by the analysis of signals originating from the protons of the ligands that are bound to the QD surface and the inclusion of an internal standard. A combined ^1^H–NMR/DOSY procedure can be a complementary method for the measurement of the NM size (hydrodynamic diameter, dH) and particle size distribution. The FTIR technique has been successfully applied to study surface effects on nanomaterials. It can be used to obtain a deep insight into the functional groups on the surface of functionalized nanomaterials. Raman spectroscopy, such as FTIR, yields information about intra- and inter-molecular vibrations and can provide additional understanding about the interactions between QDs and surface ligands. As fluorescence quenching or enhancement is strongly distance-dependent, fluorescence spectroscopy can provide information on the direct NM–ligand interface, which could shed light on the adsorption dynamics and mechanism. The binding of organic ligands on NM surfaces may change the absorption spectra of surface ligands or NMs, which makes the UV–vis spectroscopy suitable for providing complementary information. ITC is a straightforward method for measuring the association process between surface ligands and NMs in a quantitative manner, although it is time consuming and relatively high concentrations of titrant are needed. During the titration, the heat change is directly recorded and important thermodynamic parameters (including the change of enthalpy ΔH, entropy ΔS and Gibbs-free energy ΔG) and reaction parameters between QDs and surface ligands (including the binding constant and binding stoichiometry) can be calculated. The SEC method is simple, quick and relatively cheap; however, it provides no further information on the adsorption mode. The adsorption of organic ligands on NMs would change their surface charge, thus influencing the zeta (ζ) potential. Although ζ potential analyses cannot provide direct information on the surface’s binding, it served as an important auxiliary to elucidate the adsorption mechanism. Powder X-ray diffraction (XRD) is a common characterisation technique for nanoscale materials. An analysis of a sample by powder XRD provides important information that is complementary to various microscopic and spectroscopic methods, such as phase identification, sample purity, crystallite size and, in some cases, morphology. As a bulk technique, the information it provides can be correlated with microscopy data to test if microscopic observations on a small number of particles are representative of most of the sample. X-ray absorption spectroscopy is well suited for the investigation of nanomaterials, and size-dependent effects are readily detectable for nanoparticles with a size below about 10–15 nm.

Although, today, different instrumental analytical techniques can be used to study the chemical composition of QDs, their structure and the interaction with different types of ligands, only few techniques have been used for the detection and determination of QDs in real samples.

Electrochemical techniques such as voltammetry in different modes such as SWASV, SWV and ASV have been applied in different media but not in complex matrices. UV–Vis spectrophotometry and spectrofluorimetry have been proposed for the analysis of complex samples, but at the moment, no application for real samples has been reported.

Among the Atomic Spectrometry techniques, the GFAAS has been used to determine the chemical composition of the QDs, but it was not applied to quantify the presence of QDs in real samples. ICP–OES has been used to determine the liberation of cadmium from QDs in simulant fluids. In relation to ICP–MS, due to the low size of the majority of the QDs, the single particle mode (sp–ICP–MS) introduces problems in detecting this type of metal nanoparticles. Only the ICP–MS working in the single-cell mode (SC–ICP–MS) has been applied to study Cd concentrations in cells exposed to CdSe/ZnS QDs.

Due to the limitations of the current analytical techniques to perform the detection and quantification of QDs in complex samples, as well as the great concern about their liberation at the different environmental compartments and their possible toxicity for humans, it is necessary to work with a multidisciplinary perspective in the development of new analytical methods for the detection of these nanomaterials in complex real samples.

To develop new methodologies for the determination of QDs in complex media, it is mandatory to consider not only the capabilities and limitations of analytical techniques at hand but also the properties of the QDs, especially those related to their structure and surface characteristics, as has been described throughout this review.

## Figures and Tables

**Table 1:** Maximum wavelength values for fluorescence emission and visible absorption shown by CdSe as bulk material and as QDs with particle diameters in the range 1.8–7.3 nm.

CdSe	Vis. Absorption	Fluorescence Emission	
Diameter (nm)	λ_abs_ (nm)	λ_em_ (nm)	COLOUR *
Bulk material	671	713	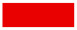
7.3	645	653	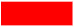
4.1	586	596	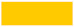
2.9	541	549	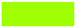
2.2	484	499	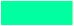
1.8	427	449	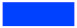

* Colours were calculated by conversion of the wavelength maximum to an RGB colour.

**Table 2:** Bandgap energy and exciton Bohr radius of some semiconductor inorganic materials, which are commonly used in QDs. Values of bulk semiconductor materials vary depending on the literature because various methods have been used to obtain them.

IV–VI QDs	Bulk Bandgap (eV) [ref.]	Bulk Exciton Bohr Radius (nm) [ref.]
PbS	0.41 [14]	18 [14]
PbSe	0.28 [14]	46 [14]
PbTe	0.31 [14]	150 [14]
II–VI QDs	Bulk bandgap (eV)	Bulk exciton Bohr radius (nm) [ref.]
ZnS	3.6 [50]	2.5 [51]
ZnSe	2.72 [52]	4.5 [52]
ZnTe	2.25 [52]	6.7 [52]
CdS	2.42 [50]	2.9 [51]
CdSe	1.73 [50]	5.6 [51]
CdTe	1.5 [14]	10 [14]
HgTe	−0.15 * [14]	80 [14]
III–V QDs	Bulk bandgap (eV)	Bulk exciton Bohr radius (nm) [ref.]
AlN	6.2 [52]	1.6 [52]
AlP	2.45 [52]	37 [52]
AlAs	2.15 [52]	41 [52]
AlSb	1.6 [52]	64 [52]
GaN	3.4 [52]	3.1 [52]
GaP	2.27 [52]	29 [52]
GaAs	1.43 [52]	12.4 [52]
GaSb	0.7 [52]	60 [52]
InN	0.65 [52]	11.4 [52]
InP	1.27 [14]/1.42 [52]	15 [14]/11 [52]
InAs	0.36 [14]	34 [14]
InSb	018 [52]	54 [52]
IV QDs	Bulk bandgap (eV)	Bulk exciton Bohr radius (nm) [ref.]
Si	1.17 [52]	4.3 [52]
Ge	0.67 [52]	11.5 [52]
II–V QDs	Bulk bandgap (eV)	Bulk exciton Bohr radius (nm) [ref.]
Cd_3_P_2_	0.55 [14]	18 [14]
Cd_3_As_2_	−0.19 * [14]	47 [14]
I–VI QDs	Bulk bandgap (eV)	Bulk exciton Bohr radius (nm) [ref.]
Ag_2_S	0.9–1.1 [14,52]	2.2 [52]
Ag_2_Se	0.15 [14,52]	2.9 [52]
I–III–VI QDs	Bulk bandgap (eV)	Bulk exciton Bohr radius (nm) [ref.]
CuInS_2_	1.53 [14,52]	4.1 [14,52]
CuInSe_2_	1.04 [14,52]	10.6 [14,52]
AgInS_2_	1.87 [52]	5.5 [52]
AgInSe_2_	1.24 [14,52]	5–6 [52]
II–II–VI QDs	Bulk bandgap (eV)	Bulk exciton Bohr radius (nm) [ref.]
CdHgTe	0–1.5 [11]	Not found

* Materials with an inverted bandgap.

**Table 3:** Relationship between the size of QDs and the number of surface atoms.

Diameter of QDs (nm)	No. of Surface Atoms	Ratio of Surface Atoms to Total Atoms (%)
10	3 × 10^4^	20
4	4 × 10^3^	40
2	2.5 × 10^2^	80
1	30	90

## Data Availability

Not applicable.
